# Metatranscriptome Data Support the Existence of Two Distinct Morphotypes in a Single Parmalean Species in Natural Environments

**DOI:** 10.1264/jsme2.ME26037

**Published:** 2026-09-18

**Authors:** Hiroto Sasaki, Hisashi Endo, Eric Pelletier, Shinya Yoshikawa, Akira Kuwata, Hiroyuki Ogata

**Affiliations:** 1 Bioinformatics Center, Institute for Chemical Research, Kyoto University, Gokasho, Uji, Kyoto, 611–0011, Japan; 2 Génomique Métabolique, Genoscope, Institut François-Jacob, CEA, CNRS, Univ Evry, Université Paris-Saclay, F-91000 Evry, France; 3 Research Federation for the Study of Global Ocean Systems Ecology and Evolution, FRA2022/Tara GOSEE, F-75016 Paris, France; 4 Faculty of Marine Science and Technology, Fukui Prefectural University, Fukui, 917–0003, Japan; 5 Shiogama Field Station, Fisheries Resources Institute, Japan Fisheries Research and Education Agency, 3–27–5 Shinhama-cho, Shiogama, Miyagi, 985–0001, Japan

**Keywords:** Parmales, metatranscriptome, morphotypes

## Abstract

Parmales, the sister clade of diatoms, includes isolates exhibiting one of two morphotypes: a silicified non-flagellated form (S-type) and a naked flagellated form (F-type). Although the alternation of these forms for a single isolate has not been formally established, these morphotypes have been hypothesized to represent alternating stages of a single parmalean population. We herein investigated the global expression patterns of S- and F-type markers by integrating parmalean environmental genomes and metatranscriptomic data from *Tara* Oceans. We detected the expression of both S- and F-type marker genes from individual environmental genomes, providing the first metatranscriptomic evidence supporting the hypothesis of a life cycle involving two morphotypes. Furthermore, the expression of F-type markers showed a broad distribution across various environmental conditions, whereas that of S-type markers was more restricted and associated with specific environmental conditions. Based on these patterns, we propose a threshold-based model in which F-type-dominated populations persist under a wide range of conditions, from oligotrophic to eutrophic environments, and a morphological switch to the S-type is triggered in a minor subset of F-type populations once environmental conditions exceed a critical threshold for growth. This study provides insights into the evolutionary acquisition of a cell type specialized for photoautotrophy in the common ancestor of parmaleans and diatoms.

Diatoms are one of the most ecologically successful groups of phytoplankton, accounting for approximately 20% of primary production in modern oceans (Armbrust, 2009). However, the drivers of their ecological success remain elusive, and comparative studies involving their close relatives are increasingly being recognized as promising approaches to address this question (Ichinomiya *et al.*, 2016).

The order Parmales (class Bolidophyceae) comprises pico-eukaryotic algae that form the sister group of diatoms (Guillou *et al.*, 1999; Ichinomiya *et al.*, 2011). Two morphological forms were originally described: a silicified form surrounded by silica plates (S-type) (Booth and Marchant, 1987) and a naked flagellated form possessing two unequal flagella (F-type) (Guillou *et al.*, 1999). A novel silicified morphotype covered with distinct silica scales, referred to as ‘Scaly parma’, was recently identified within the basal lineage of Parmales (Ban *et al.*, 2023). This discovery led to‍ ‍the recent establishment of the order Lepidoparmales to‍ ‍accommodate this lineage (Kamakura *et al.*, 2025). Although these two morphological forms were historically regarded as distinct lineages, molecular phylogenetic anal­yses revealed their close relationship (Ichinomiya *et al.*, 2011). Consequently, S- and F-type algae—originally classified separately—were both reclassified into the single order Parmales within the class Bolidophyceae (Ichinomiya *et al.*, 2016). Ecologically, S-type cells are the most abundant in polar and subarctic regions (Booth *et al.*, 1980; Ichinomiya and Kuwata, 2015; Ichinomiya *et al.*, 2016), while F-type populations were originally isolated from the equatorial Pacific Ocean and the Mediterranean Sea (Guillou *et al.*, 1999). Despite these habitat preferences based on isolation, microscopic observations and molecular studies indicate that the algae in Bolidophyceae are distributed globally (Silver *et al.*, 1980; Ban *et al.*, 2024) and their morphological forms display different distributions across depth and seasons (Kuwata *et al.*, 2018). These algae are referred to as parmaleans in this study.

The close evolutionary relationship between S- and F-type parmaleans suggests that these morphotypes correspond to different life stages of a single organism. Mann and Marchant (1989) hypothesized that the life cycle of ancestral diatoms consisted of haploid flagellated cells and diploid silicified zygotes akin to that of the early branching centric diatoms. This type of life cycle is also known for other algae, such as the haptophyte *Gephyrocapsa huxleyi* (Green *et al.*, 1996). Given the evolutionary relatedness between S- and F-type parmalean strains and their sister relationship to diatoms, Ichinomiya *et al.* (2011) hypothesized that Parmales possess a life cycle that switches between a diploid silicified form and a haploid flagellated form. This hypothesis was supported by several lines of evidence. A previous study on a flagellated strain revealed a low level of heterozygosity, suggesting the haploid status of the genome for this strain (Kessenich *et al.*, 2014). Furthermore, the flagellated strain *Triparma* sp. RCC1657 was found to belong to a clade of silicified forms based on SSU rRNA gene sequences, despite its morphology (Ichinomiya *et al.*, 2016). In addition, a genome sequencing study on silicified strains revealed the presence of genes related to the flagellated form in their genomes, which led to the hypothesis that putative life cycle switching corresponds to an alternation between a silicified photoautotrophic stage and a naked flagellated phago-mixotrophic stage (Ban *et al.*, 2023). Specifically, Ban *et al.* (2024) identified genes associated with phagocytosis in parmalean genomes—genes that have been lost in diatoms—and proposed that this mixotrophic strategy facilitates their adaptation to varying nutrient environments. Furthermore, a recent global metabarcoding study consistently revealed the cosmopolitan distributions of parmaleans, which may be explained by their life cycle strategy (Ban *et al.*, 2024). However, to the best of our knowledge, life cycle switches between the two morphological forms in a single parmalean have not been formally established, although Ichinomiya *et al.* (2016) mentioned the appearance of flagellated cells in cultures of silicified parmaleans (Ichinomiya *et al.*, 2016). Therefore, the hypothesis on the life cycle and genome state of parmaleans remains to be tested.

Large-scale environmental omics resources provide a powerful framework to assess this hypothesis. The *Tara* Oceans expedition generated global-scale metagenomic and metatranscriptomic datasets for surface and mesopelagic plankton (Sunagawa *et al.*, 2020). Among its key outputs, the Marine Atlas of *Tara* Oceans Unigenes (MATOU) provides a comprehensive catalog of eukaryotic gene expression in the marine environment (Carradec *et al.*, 2018; Pierella Karlusich *et al.*, 2025).

In the present study, we exami­ned the hypothesis that the S- and F-type forms represent distinct life-cycle stages of a single parmalean. Under this hypothesis, genes specific to the S- or F-type forms are expressed from a single parmalean, possibly in different geographic locations or environmental conditions. Specifically, we aimed to clarify whether the genes related to the silicified or flagellated forms are expressed from individual parmalean populations (at the species level) in the ocean and if the geographic distributions of their expression are environmentally regulated. To achieve this, we selected marker genes representing the two distinct morphological forms based on differential gene expression between cultured strains according to publicly available transcriptome data. We then exami­ned the presence of these genes in two parmalean metagenome-assembled genomes (MAGs) derived from *Tara* Oceans metagenomic data (Delmont *et al.*, 2022). By integrating these MAGs with the *Tara* Oceans metagenome and MATOU dataset, we characterized the global expression patterns of these marker genes and correlated them with environmental parameters.

## Materials and Methods

### Transcriptome data

We analyzed publicly available RNA-seq data from S- and F-‍type parmalean strains. Regarding the S-type strain, raw reads of *Triparma laevis* f. *inornata* strain NIES-2565 (six samples: three silicate-rich [concentration of Si, 50 μM] and three silicate-deficient [0.23 μM] replicates) were retrieved from the NCBI Sequence Read Archive (SRA) (BioProject PRJDB4267). Both conditions were treated as the S-type in the present study because the overall expression profiles were quasi-indistinguishable between conditions (Fig. S1), even though a previous study reported that the strain generally lost its silica plates under silicate-deficient conditions (Yamada *et al.*, 2014). Data for four F-‍type strains (*Triparma eleuthera* CCMP1866, *Triparma* sp. RCC1657, RCC208, and RCC2347) were obtained from the Marine Microbial Eukaryote Transcriptome Sequencing Project (MMETSP) database (Keeling *et al.*, 2014).

Reads were quality-filtered with fastp v0.24.0 (Chen *et al.*, 2018), rRNA-depleted with SortMeRNA v4.3.7 (Kopylova *et al.*, 2012) using the PR2 database v5.1.0 (Guillou *et al.*, 2013), assembled *de novo* with Trinity v2.15.2 (Grabherr *et al.*, 2011), and redundancy-reduced using the EvidentialGene pipeline (https://sourceforge.net/projects/evidentialgene/) to obtain non-redundant coding sequence (CDS) and protein sequences for each strain.

### Gene expression quantification and completeness assessment

To quantify gene expression levels, the quality-filtered RNA-seq reads were mapped back to the constructed non-redundant reference gene catalogs using Salmon (version 1.10.3) (Patro *et al.*, 2017) in the quasi-mapping mode with default parameters. This provided transcript abundance estimates for each sample, which were subsequently used for a downstream differential expression anal­ysis.

To assess the completeness of the assembled transcriptomes, we performed a BUSCO v5.8.3 anal­ysis (Manni *et al.*, 2021). The non-redundant predicted protein sequences from the five transcriptomes were evaluated against the stramenopile_odb12 lineage dataset. The same BUSCO assessment was also performed on the coding sequences of eight publicly available parmalean genomes for comparative purposes.

### Orthology inference

To investigate evolutionary relationships and identify core gene sets, we performed an orthology anal­ysis using OrthoFinder v2.3.8 (Emms and Kelly, 2019). The input dataset included the predicted protein sequences from (1) the five assembled transcriptomes (one S-type and four F-types described above), (2) eight publicly available parmalean genomes, (3) two parmalean MAGs retrieved from the *Tara* Oceans dataset: TARA_ARC_108_MAG_00221 (ARC-MAG) and TARA_PON_109_MAG_00217 (PON-MAG), and (4)‍ ‍three diatom genomes (*Thalassiosira pseudonana*, *Pseudo-nitzschia multistriata*, and *Fragilariopsis cylindrus*) included as outgroups.

### Phylogenetic anal­ysis

Based on OrthoFinder results, single-copy orthologous genes conserved across all genomes and transcriptomes were extracted to reconstruct the phylogeny. The sequences were concatenated and aligned, and a maximum likelihood phylogenetic tree was constructed using IQ-TREE 3.0.1 (Wong *et al.*, 2026). ModelFinder (Kalyaanamoorthy *et al.*, 2017) was used to select the best-fit substitution model, and branch support was assessed using ultrafast bootstrapping (Hoang *et al.*, 2018).

### Identification of morphotype-specific marker genes

We extracted orthogroups conserved across all eight Parmales reference genomes and filtered for genes with high expression levels (log counts per million [CPM] >5) from the OrthoFinder output. Marker genes were then classified based on their exclusive expression into S- or F-type cultures. This conservative classification strategy was adopted to minimize false positives arising from inter-strain differences.

### Metagenomic and metatranscriptomic mapping to MAGs

Morphotype-specific marker gene sequences were extracted from the two parmalean MAGs (ARC-MAG and PON-MAG) based on orthogroup assignments. Metagenomic and metatranscriptomic reads were mapped to these MAG-derived marker genes using bwa-mem2 v2.2.1 (Vasimuddin *et al.*, 2019) with default parameters. The resulting alignments were sorted and deduplicated using samtools v1.18 (Danecek *et al.*, 2021). To ensure high-confidence mapping (Carradec *et al.*, 2018), reads were filtered using bamFilters v1.10.2 (https://github.com/institut-de-genomique/bamFilters) with a minimum sequence identity of 95% and minimum alignment length coverage of 90%. Mapped reads were extracted, and raw read counts were aggregated per sample to construct a count matrix.

### Clustering and visualization of expression patterns

A Bray-Curtis dissimilarity matrix was calculated from the marker gene expression matrix using the vegan package v2.7-1 in R v4.5.1, and hierarchical clustering was performed using the Ward.D2 method. Results were visualized as heatmaps with clustering dendrograms using the pheatmap package v1.0.13. Expression data were also integrated with *Tara* Oceans environmental metadata.

### Environmental modeling

To investigate the relationships between morphotype expression and environmental factors, we employed generalized additive models (GAMs) using the mgcv package v1.9.3. In this modeling framework, the response variable was defined as a normalized expression index calculated using log_10_-transformed reads with a pseudo count of 1. Specifically, the index for each type (F or S) was calculated as:

$$\text{Index}=\frac{\log_{10}\left(T_{type}+1\right)}{\log_{10}\left(G_{total}+1\right)}$$

where *T_type_* represents metatranscriptomic read counts for type-specific marker genes (F- or S-type), and *G_total_* represents the sum of metagenomic read counts for both F- and S-type genes. Samples with low gene abundance (*G_total_* <10) were excluded from the anal­ysis. Explanatory variables included dissolved inorganic carbon (DIC), nutrient concentrations (PO_4_, Si, and NO_2_+NO_3_), and physical parameters (temperature, salinity, and density). Sampling depth (surface [SRF] or deep chlo­rophyll maximum [DCM]) was included as a categorical factor. Prior to the anal­ysis, we confirmed that the variance inflation factor (VIF) for all continuous predictors was less than 10. The model assumed a Tweedie error distribution with a log link function, and parameter estimations were performed using REML with the “select=TRUE” option enabled. This applies additional shrinkage, allowing the model to shrink non-contributing smooth terms toward zero, effectively removing them.

## Results

### *De novo* transcriptome assembly of five cultured Parmales strains

To examine possible morphotype-specific genes in parmaleans, we generated a *de novo* RNA-seq assembly for five strains: the silicified strain NIES-2565 (*n*=6) and four flagellated strains (CCMP1866, RCC1657, RCC208, and RCC2347; *n*=1 each), yielding 19,000–30,000 transcripts per strain (Table 1). The BUSCO anal­ysis (stramenopiles_odb12) confirmed completeness scores exceeding 60% for all assemblies, which was similar to the reference complete genomes.

### Identification of morphotype-specific markers

Prior to marker gene selection, we investigated whether the silicate-rich and silicate-deficient culture conditions of NIES-2565 produced distinct expression profiles. A principal coordinates anal­ysis (PCoA) based on the Bray-Curtis dissimilarity of ortholog-mapped read counts across all ten samples revealed that the first principal coordinate (PCoA1), accounting for 63.3% of the total variance, clearly separated the four F-type strains from all six NIES-2565 samples. The silicate-rich and silicate-deficient replicates of NIES-2565 were intermingled along this axis, indicating that culture conditions had a negligible effect on global expression patterns relative to the morphotype difference. Therefore, all six NIES-2565 samples were treated as S-type replicates in subsequent marker gene selection.

To identify genes specific to the S- or F-type forms, we conducted an ortholog anal­ysis of 18 strains (five transcriptomes, eight parmalean genomes, and five diatom outgroups) and yielded 25,752 orthogroups. Of the 5,638 orthogroups conserved across all eight reference genomes (“Parmales core gene set”), 39 were expressed exclusively in silicified cultures and 652 exclusively in flagellated cultures. The application of a stringent expression threshold (logCPM ≥5.0) resulted in 30 S-type and 180 F-type marker genes (Fig. 1). Functional annotation confirmed that S-type markers included the silicon transporter (SIT) and F-type markers contained intraflagellar transport (IFT) genes, validating their correspondence to the silica cell wall and flagella, respectively. ARC-MAG and PON-MAG, the two parmalean MAGs analyzed in this study, encoded 92% (166/181) of F-type markers and 73% (22/30) of S-type markers, while PON-MAG encoded 81% (139/181) and 90% (26/30), respectively.

### Phylogenetic relationships and MAG placement

To ensure that our MAGs were representatives of the Parmales known diversity, we inferred a phylogenetic tree based on 75 concatenated single-copy orthologous genes, which reproduced the established classification of Parmales with full bootstrap support (Fig. 2). The two MAGs were placed in distinct clades: ARC-MAG in the Lepidoparmales group (Clade I) and PON-MAG in the *Triparma* group (Clade IV), representing two phylogenetically distant lineages. An average nucleotide identity (ANI) anal­ysis further confirmed the close affinity of ARC-MAG to the *Lepidoparma frigida* reference genome (99.4% identity).

### *In situ* expression of both morphotype markers from individual MAGs

To investigate the geographic distributions of S- and F-type markers, we mapped *Tara* Oceans metagenomic and metatranscriptomic reads to the two MAGs. The metagenomic abundance (*i.e.*, “presence”) of S- and F-type markers strongly correlated within each MAG, confirming stable genomic copy number ratios (Fig. S2, Table 2). In contrast, metatranscriptomic abundance (*i.e.*, “activity”) markedly varied across samples.

The mapping of *Tara* Oceans metatranscriptomic reads to the two MAGs revealed that all of the S- and F-type marker genes encoded in the MAGs were transcriptionally active at least in one sample. The hierarchical clustering of expression profiles showed that the S- and F-type markers formed distinct clusters within each MAG (Fig. 3), indicating that the two marker sets were independently regulated.

Spatially, metagenomic distributions were synchronized between markers, whereas transcriptomic patterns diverged (Fig. 4). ARC-MAG transcription was restricted to polar regions, with F-type markers being up-regulated in the Atlantic inflow at the DCM layer (Fig. 4d). Conversely, PON-MAG transcripts were concentrated in low-to-middle latitudes, showing higher abundance in the DCM (Fig. 4h) than in surface waters (Fig. 4g). While F-type transcripts remained prevalent, S-type expression markedly increased within the DCM, particularly in the Eastern Pacific.

### Distinct scaling regimes of morphotype-specific gene expression

The relationship between total gene abundance and S-type transcript abundance revealed two distinct scaling regimes (Fig. 5). In the high-expression regime (Group 1), S-type transcripts scaled near-linearly with gene abundance (slopes: 1.18±0.12 for ARC-MAG, 0.90±0.08 for PON-MAG; R^2^=0.82 and 0.86, respectively). In the basal regime (Group 2), scaling was markedly weaker (slopes: 0.54±0.07 and 0.28±0.07; R^2^=0.24 and 0.08), with S-type transcript levels remaining low and stochastic regardless of gene abundance. In contrast, F-type transcript abundance scaled uniformly with gene abundance without exhibiting such regime separation (slopes: 0.80±0.09 and 0.87±0.06; R^2^=0.28 and 0.54).

### Environmental drivers of morphotype-specific gene expression

We further exami­ned the environmental parameters potentially affecting the expression profiles of morphotype marker genes. GAMs revealed contrasting environmental dependencies between the two marker types (Fig. 6 and S3). Notably, S-type models explained more deviance (48.8% for ARC-MAG, 26.0% for PON-MAG) than F-type models (19.7% and 5.8%), indicating the closer coupling of S-type expression to abiotic variables. In ARC-MAG (*n*=146), S-‍type expression correlated with PO_4_, Si, temperature, salinity, and DIC, whereas F-type expression responded primarily to temperature and NO_2_+NO_3_. In PON-MAG (*n*=142), temperature was the dominant driver for both types, with PO_4_ additionally affecting S-type expression and NO_2_+NO_3_ impacting F-type expression. Sampling depth was not significant in any model.

## Discussion

In the present study, we detected the expression of marker genes for both the S- and F-type forms from individual parmalean MAGs. While Ban *et al.* (2023) previously demonstrated that parmaleans (S-type) possessed the genomic repertoire for these distinctive functions, the results obtained herein indicate, for the first time, that environmental parmalean populations (at the species level) manifest gene expression for both morphotypes. The hypothesis that these two forms belong to the same species was primarily inferred from molecular phylogenies (Ichinomiya *et al.*, 2016). Our results support the view that these dual genetic traits are functional in actual marine environments, at least within the lineages represented by the two analyzed MAGs.

The global distribution patterns of the two MAGs align well with the biogeography of parmalean clades elucidated by Ban *et al.* (2024): ARC-MAG was predominantly found‍ ‍in polar and subarctic regions, mirroring Clade I (Lepidoparmales), while PON-MAG was detected in tropical and temperate regions, corresponding to the warmer-water populations within the ubiquitous Clade IV.

A notable observation was the contrasting distribution patterns between S- and F-type marker genes at the transcriptomic level, despite their stable genomic ratios across all sites. This genomic stability indicates that the geographically distinct expression patterns are driven by differential transcriptional activity rather than differences in intra-species genetic variations. This result offers a coherent explanation for the long-standing discrepancy between the global ubiquity reported in amplicon sequence variant (ASV)-based surveys and the restricted range of silicified strain isolations: populations in warmer waters are likely represented primarily by the flagellated form.

The marked variation in metatranscriptomic abundance across samples suggests that marker expression is dynamically regulated by local environmental conditions; specifically, the response of S-type expression appears to be governed by specific environmental parameters. Our scaling anal­ysis revealed two distinct regimes of S-type gene expression (Fig. 5). In the high expression regime, S-type gene expression is proportional to relative genome abundance within the planktonic community. This, together with environmental modeling (Fig. 6), suggests that conditions favorable for biomass increases trigger active silicification. In contrast, F-type expression is constitutively maintained, allowing the organism to tolerate a wide range of environments. This interpretation aligns with culture experiments showing that the S-type is cold-adapted with limited growth above 15°C (Ichinomiya *et al.*, 2013), whereas the F-type exhibits broader thermal tolerance (Stawiarski *et al.*, 2016). Therefore, the “ubiquitous” distribution of Clade IV in ASV studies likely reflects the capacity of the F-type to inhabit warmer waters, while the S-type is confined to niches satisfying specific growth conditions.

This niche separation is illustrated by PON-MAG in tropical regions, where S-type expression was markedly lower in surface waters than in the DCM, consistent with microscopic observations showing that silicified cells were restricted to deep photic layers (Fujita and Jordan, 2017). While some studies reported the sporadic occurrence of specific silicified species in tropical and subtropical surface waters, these findings generally represent low abundance up to the order of 10^4^ cells L^–1^ (Kosman *et al.*, 1993; Bravo-Sierra and Hernández-Becerril, 2003). This contrasts with the markedly higher concentrations of silicified cells in subpolar and polar regions, where S-type parmalean cell densities typically reach the order of 10^5^ cells L^–1^ (Silver *et al.*, 1980; Booth and Marchant, 1987; Ichinomiya and Kuwata, 2015). Therefore, historical reports of parmalean scarcity in tropical surface waters may reflect suppressed silicification below detection limits. In contrast, F-type markers were robustly detected in these surface layers, suggesting that the flagellated form inhabits this warm niche, remaining cryptic to morphological observations. The presence of the F-type in these typically oligotrophic surface waters may reflect differences in trophic strategies. Ban *et al.* (2023) hypothesized dimorphic trophic modes: primarily autotrophic for the S-type and phago-mixotrophic for the F-type. Our results are consistent with this hypothesis because S-type markers prevailed in the nutrient-rich DCM while F-type markers dominated in nutrient-depleted surface waters where bacterial grazing may sustain a mixotroph. Nevertheless, since the flagellated form is hypothesized to function as a gamete, its surface presence may partly reflect life-cycle transitions.

Our GAM anal­ysis provides statistical support for these divergent strategies. S-type models demonstrated markedly higher explanatory power than F-type models, indicating that S-type expression is strongly coupled to abiotic variables while F-type expression is largely decoupled from measured physicochemical parameters. This contrast reinforces the hypothesis of distinct trophic modes: strict autotrophy may be directly regulated by inorganic nutrients, while a mixotrophic F-type driven by prey availability may not be captured by standard environmental metadata. Among the specific drivers, S-type expression showed consistent positive relationships with PO_4_ in both MAGs. Regarding ARC-MAG, the additional relationship with lower salinity suggests an affinity for river-influenced, nutrient-rich Arctic waters, while the negative relationship with Si may reflect a rapid drawdown by actively silicifying populations.

Building on these contrasting environmental dependencies, we propose a threshold-based model for the morphological switch in parmaleans. Our scaling anal­ysis revealed that S-type accumulation consistently occurred above a minimum abundance threshold, suggesting that the transition from flagellated to silicified phases is not gradual, but requires conditions sufficient to sustain stable growth. Below this threshold—as observed in oligotrophic surface waters—the community appears to persist primarily in the F-type form, the mixotrophic capacity of which enables survival in nutrient-poor environments where strict autotrophy may be untenable. Under such low-abundance conditions, only the F-type may proliferate due to its potentially higher growth rate (Kuwata *et al.*, 2018). As environmental conditions improve to allow the overall population to expand, this increase in biomass triggers the morphological switch to the S-type. Crucially, even under these favorable growth conditions, F-type transcript abundance is scaled with overall gene abundance (Fig. 5). This suggests that F-type cells remain to increase and constitute the majority of the total population. This model reconciles the cryptic, F-type-dominated populations in warm oligotrophic waters with the conspicuous blooms of silicified cells reported in nutrient-rich regions (Ban *et al.*, 2024).

Ban *et al.* (2023) proposed that during the early stage of evolution following their divergence from the parmalean lineage, diatoms became specialized for photoautotrophy by discarding their phagotrophic capacity and acquiring genes associated with autotrophic adaptations. Under growth-favorable conditions, diatoms may rapidly proliferate. Similarly, the growth of parmalean S-type cells appears to be triggered by environmental factors. Therefore, the shared capacity of these silicified cells to respond to favorable environmental conditions may be a trait of silicified cells of their common ancestor. Since flagellated cells are considered to be more ancestral than silicified cells in ochrophyte lineages (Mann and Marchant, 1989), the silicified form in the common ancestor of diatoms and parmaleans may have emerged as an adaptation to nutrient-rich conditions.

There are several limitations in the present study. The model proposed above must be interpreted with caution. The low abundance of Parmales (<0.2% of the community [Ichinomiya *et al.*, 2016; Kuwata *et al.*, 2018]) limited the number of mapped reads, and missing environmental metadata necessitated sample exclusions that may have introduced a bias. Furthermore, although the selected marker genes were conserved in all available parmalean genomes, the transcriptomic data for the S-type were derived from only a single cultured strain (NIES-2565). This may limit the capacity to characterize the physiological states of diverse parmaleans. To confirm the life-cycle model, it is necessary to move beyond static distributional snapshots. While culture-based direct observations of S–F transitions remain the ultimate goal, field-based temporal data may offer an important alternative. Specifically, this may involve collecting time-series metatranscriptomic data from environments with low levels of parmalean diversity, allowing for the tracking of sequential changes in S- and F-type gene expression levels. Priority needs to be given to high-abundance regions absent from our dataset, such as the Western North Pacific (Oyashio) and the east coast of Australia, where Clade II blooms are known to occur (Ban *et al.*, 2024). The integration of these targeted environmental profiling efforts will be an effective approach to elucidate the environmental drivers of morphological switching in this unique lineage.

## Citation

Sasaki, H., Endo, H., Pelletier, E., Yoshikawa, S., Kuwata, A., and Ogata, H. (2026) Metatranscriptome Data Support the Existence of Two Distinct Morphotypes in a Single Parmalean Species in Natural Environments. *Microbes Environ ***41**: ME26037.

https://doi.org/10.1264/jsme2.ME26037

## Supplementary Material

Supplementary Material

## Figures and Tables

**Fig. 1.**
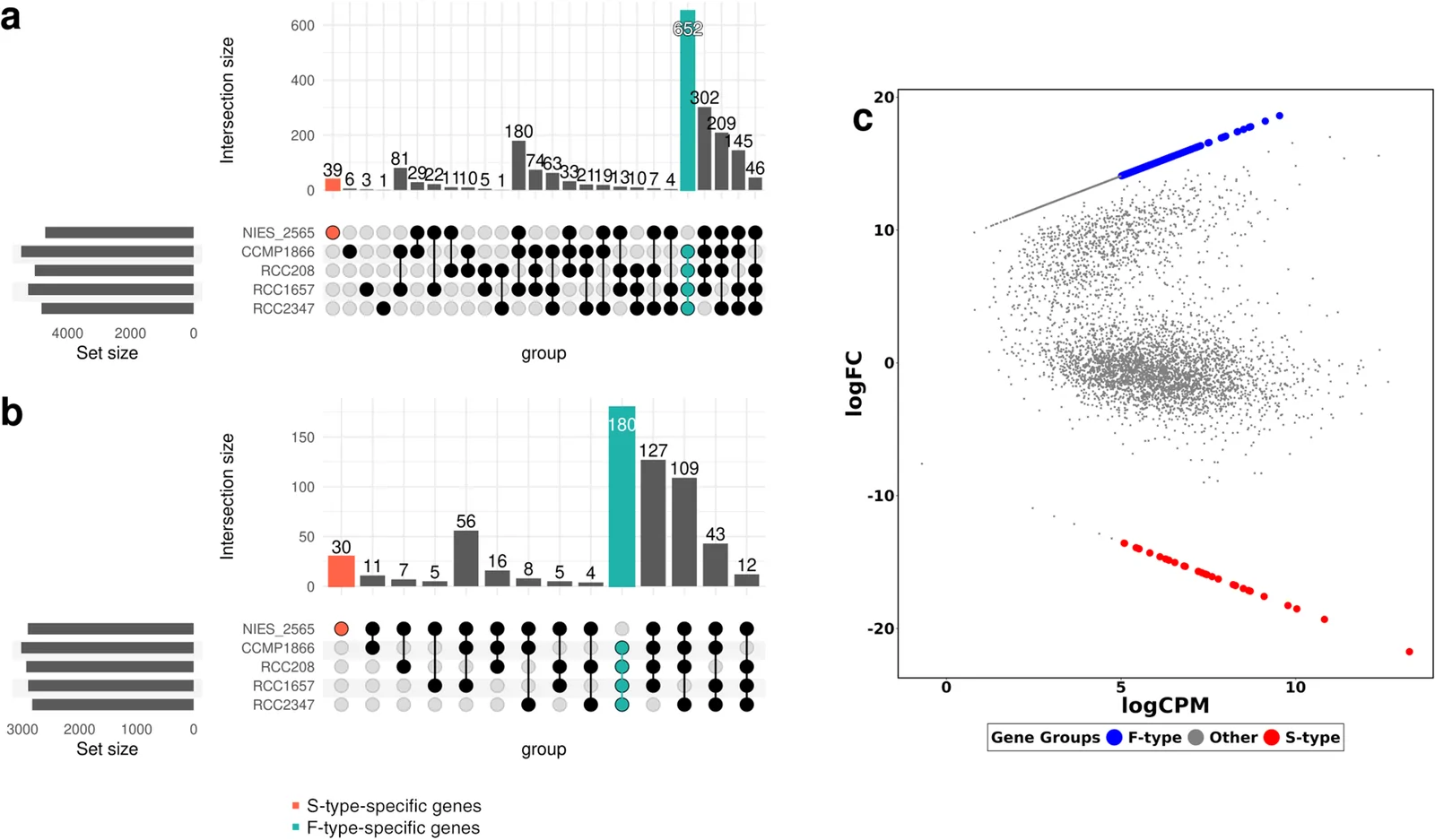
Genes specific to Parmales morphotypes. (a, b) UpSet plots illustrating the distribution of orthologs among different Parmales strains with transcriptome data. The numbers above the vertical bars (Intersection Size) indicate the number of orthologs shared by a specific combination of strains. The horizontal bars on the left (Set Size) show the total number of orthologs within each strain. The red bar corresponds to the orthologs unique to the S-type transcriptome, while the teal bar represents the orthologs identified in all F-type transcriptomes, but not in the S-type. Panel (a) includes all orthologs that were found to be universally conserved in eight Parmales strains with genomic sequence data. In panel (b), only orthologs with an expression level of logCPM ≥5.0 are shown. (c) MA plot showing genes specifically expressed in individual morphotypes. Red points indicate orthologs selected as being specific to the S-type and blue ones as those being specific to the F-type.

**Fig. 2.**
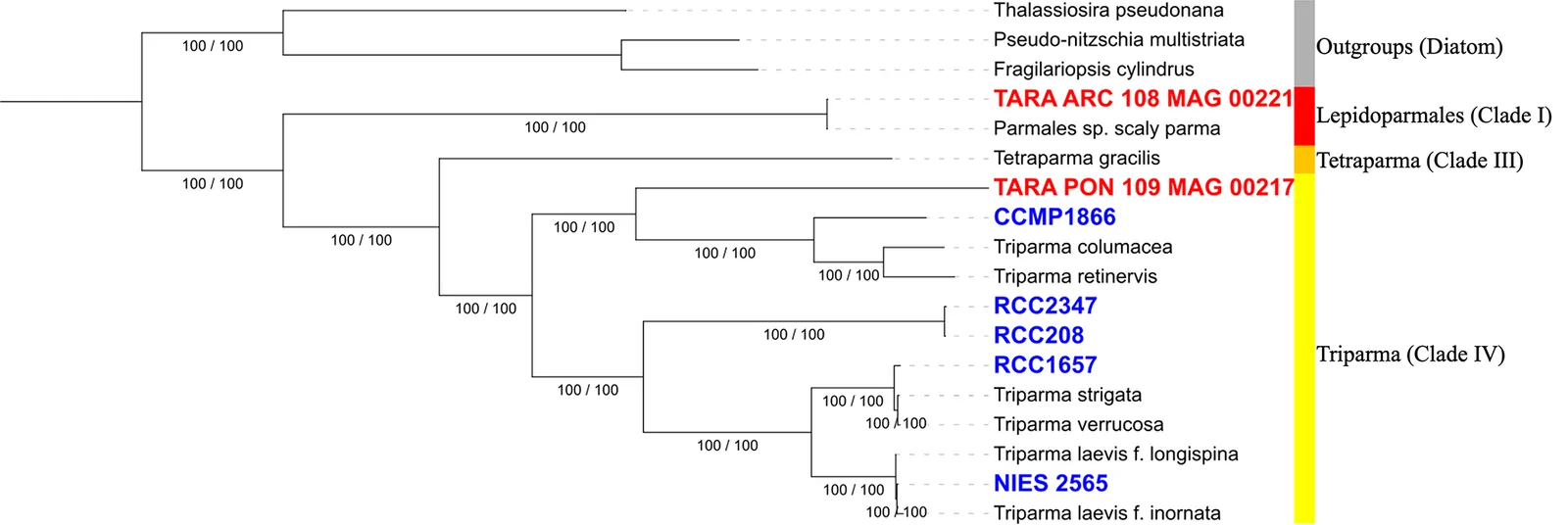
Phylogenetic relationships inferred from concatenated single-copy genes. Maximum likelihood phylogenetic tree of concatenated alignments of single-copy orthologous genes. IDs shown in blue represent the phylogenetic placement of assemblies derived from RNA-seq data. IDs shown in red indicate the positions of parmalean metagenome-assembled genomes (MAGs) from the *Tara* Oceans dataset. Nodal support is provided by 1,000 ultrafast bootstrap replicates (UFB, first value) and SH-aLRT (SH, second value).

**Fig. 3.**
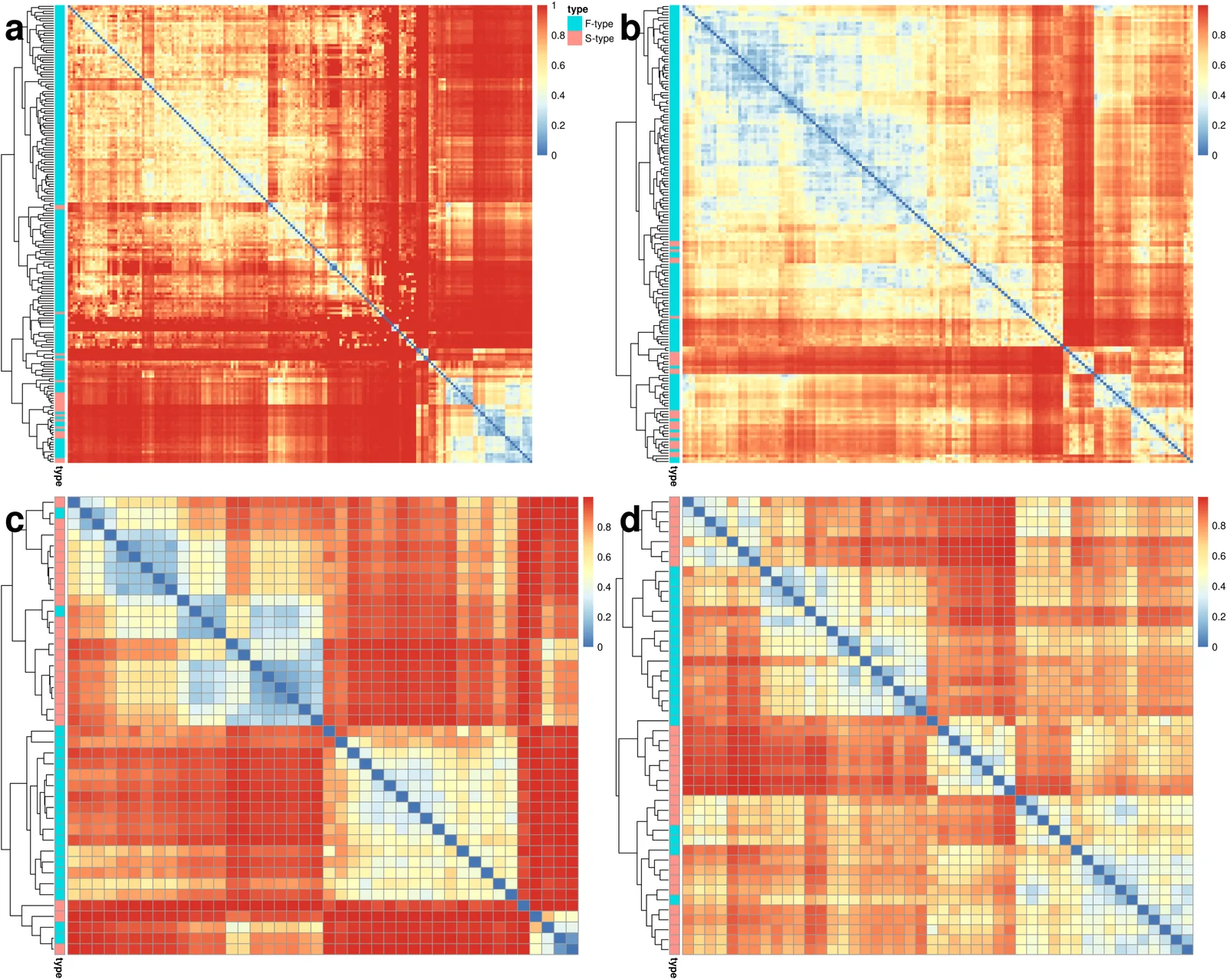
Expression profiles of S- and F-type marker genes in two parmalean MAGs. Heatmaps displaying the similarity of expression patterns among S- and F-type marker genes. The Bray-Curtis dissimilarity among marker genes was calculated based on the read counts of genes among samples. The color scale indicates the dissimilarity of expression patterns across orthologs. The annotation bars on the left indicate the morphological types: green for F-type and red for S-type. (a, b) show the clustering of all marker genes for the Arctic MAG (a) and Pacific Ocean North MAG (b). (c, d) show the clustering of the 50 most highly expressed F-type markers and all S-type markers for the Arctic MAG (c) and Pacific Ocean North MAG (d).

**Fig. 4.**
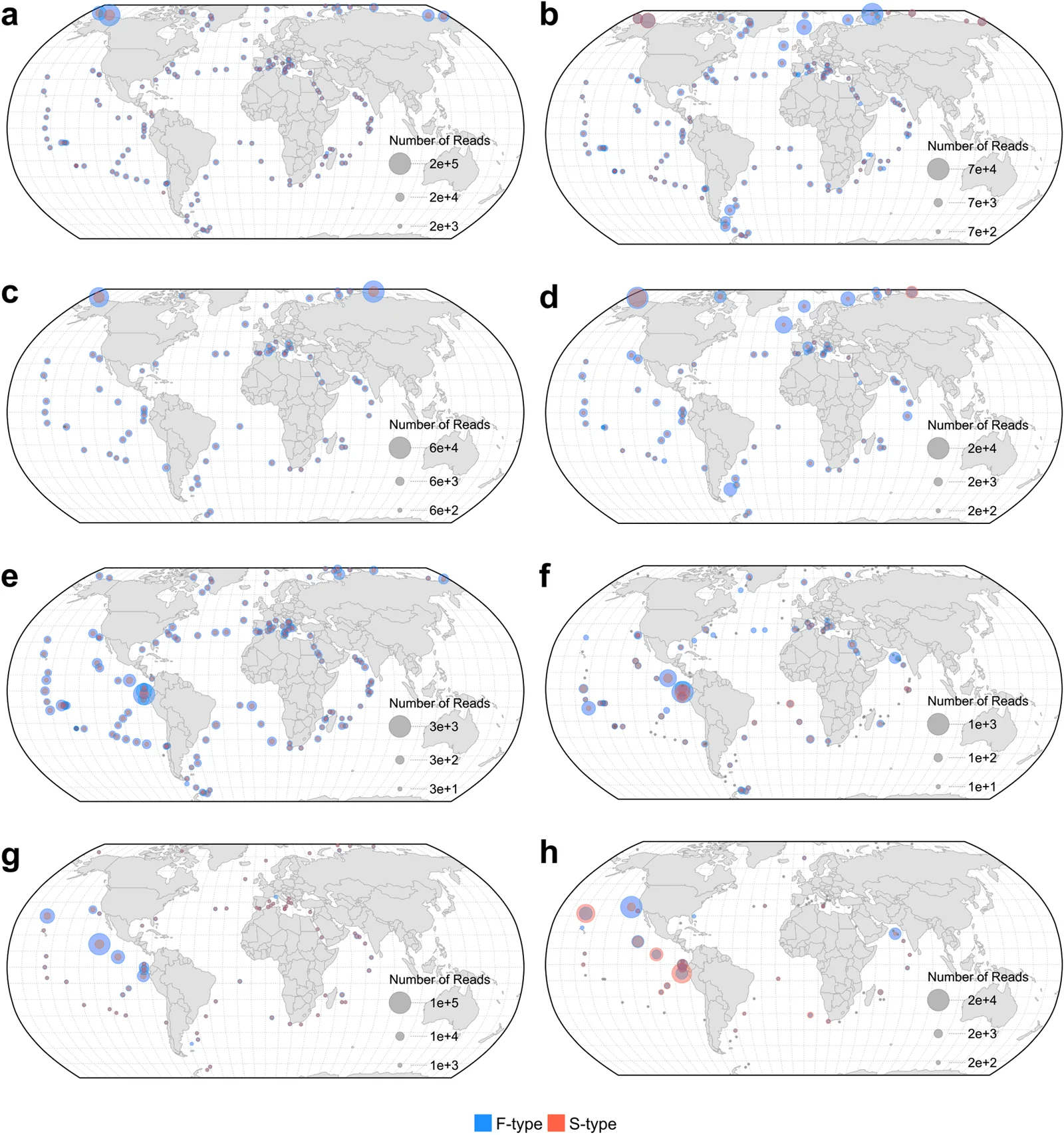
Global distribution and expression patterns of S- and F-type marker genes for two parmalean MAGs. The maps show read counts from metagenomes (left column: a, c, e, g) and metatranscriptomes (right column: b, d, f, h). Bubble size is proportional to the number of reads, and color indicates the marker type (blue: F-type; red: S-type). Sites with read counts <10 are shown as dots. Samples are organized by MAG and depth layer: (a, b) ARC-MAG in surface waters; (c, d) ARC-MAG in the deep chlo­rophyll maximum (DCM) layer; (e, f) PON-MAG in surface waters; (g, h) PON-MAG in the DCM layer.

**Fig. 5.**
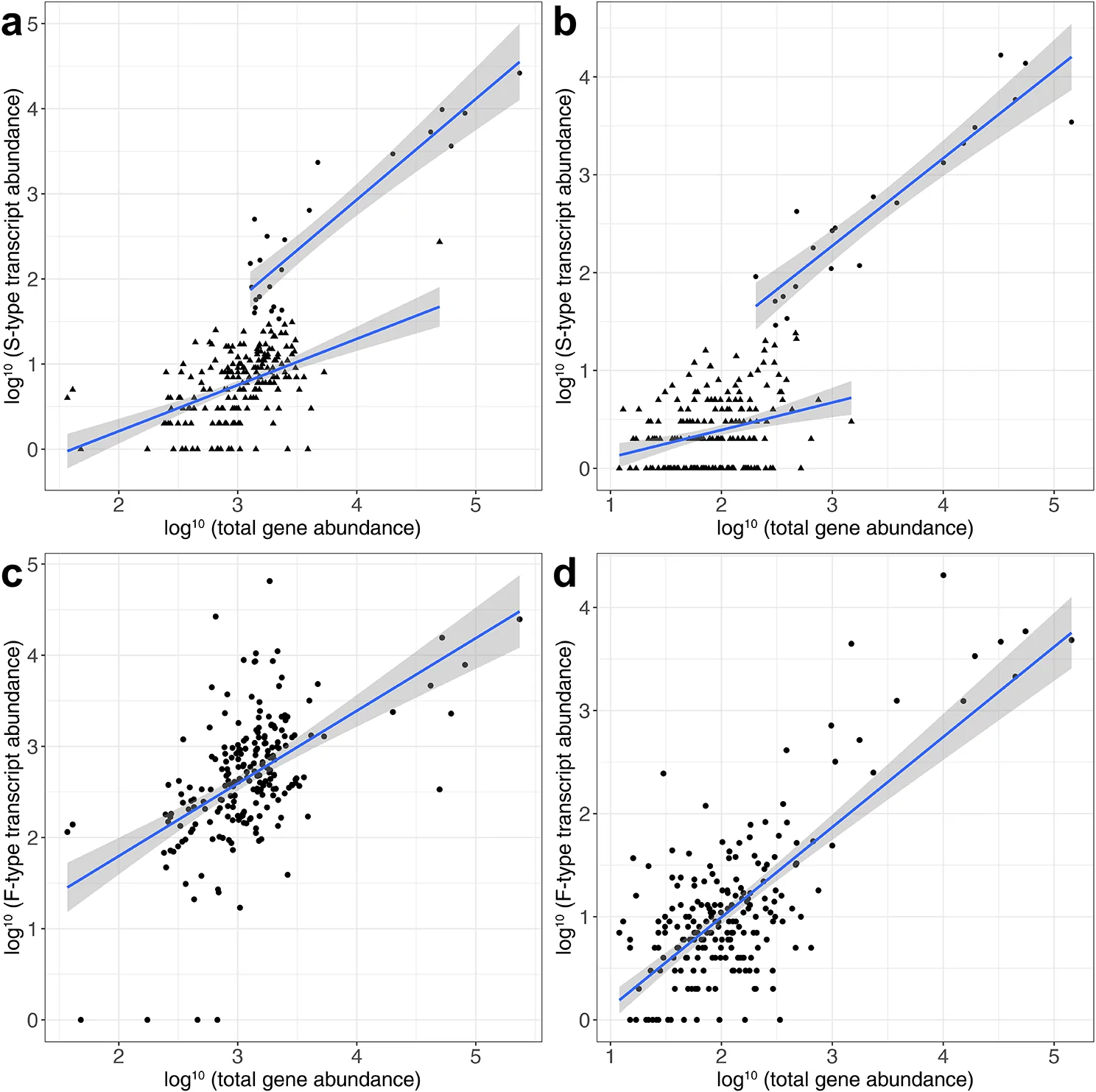
Scaling relationship between total gene abundance and morphotype-specific transcript abundance in ARC-MAG and PON-MAG. Log-log scatter plots showing the relationship between log10-transformed total gene abundance (metaG) and marker transcript abundance (metaT). (a, b) S-type markers for ARC-MAG and PON-MAG, respectively. Data points are divided into two groups based on the following conditions that were selected by a visual inspection of the bimodal distribution: a subset showing a proportional response (Group 1, circles) and the remaining points (Group 2, triangles). Specifically, Group 1 was defined based on (*X* + *Y* ≥ 3.5) ∧ (*Y* ≥ 1.5) ∧ (*X* − *Y* < 2) for ARC_MAG, and (*X* + *Y* ≥ 3.8) ∧ (*Y* ≥ 1.4) for PON-MAG, where *X* = log_10_(*metaG*) and *Y* = log_10_(*metaT*). (c, d) F-type markers for ARC-MAG and PON-MAG, respectively, showing uniform scaling without distinct regimes. Blue lines indicate linear regression fits with 95% confidence intervals (grey shading).

**Fig. 6.**
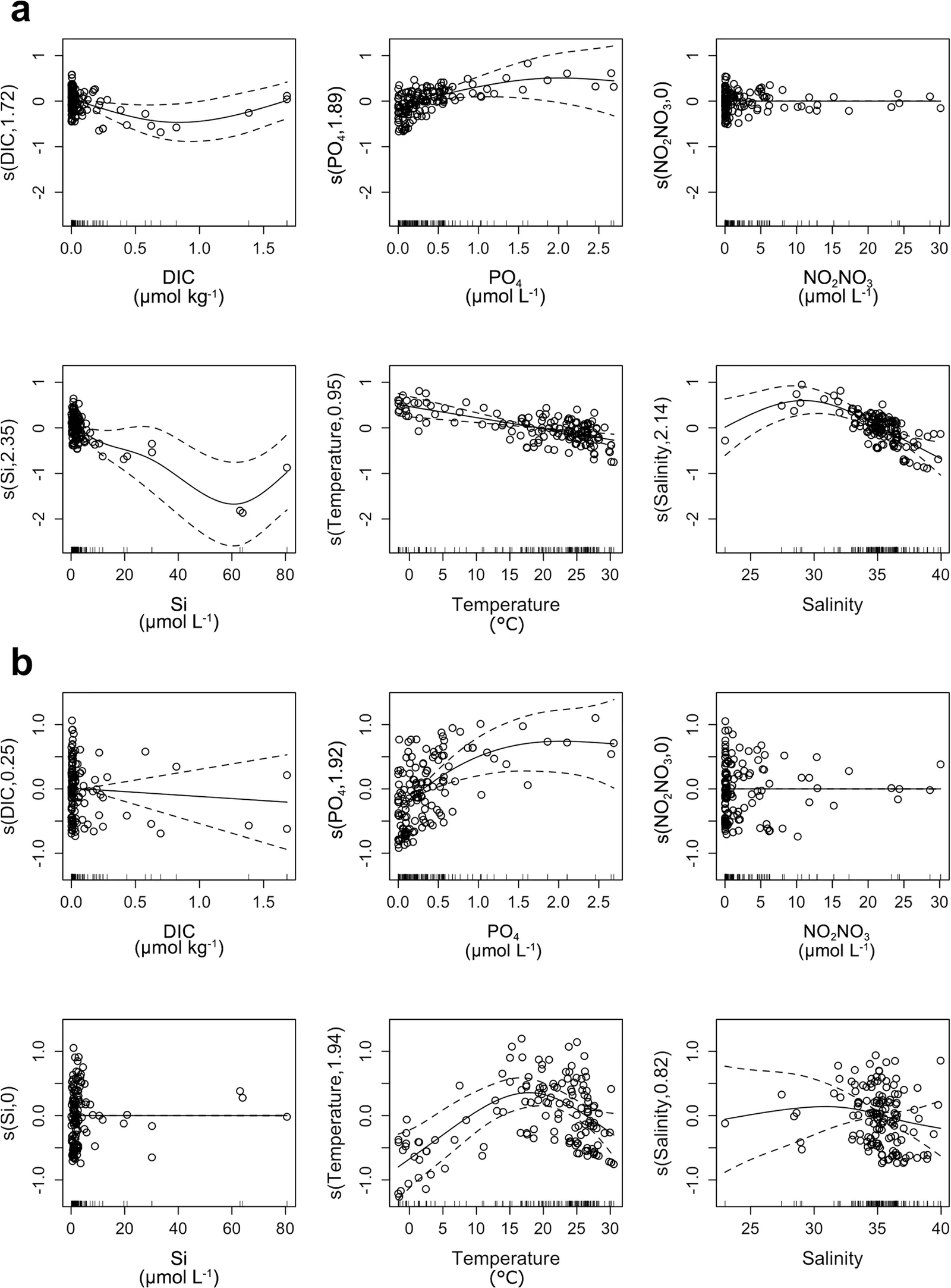
Generalized additive model (GAM) regression anal­ysis of S-type-specific expression variations based on environmental factors. Panels show the partial effects of environmental variables on expression indices for the ARC-MAG dataset (*n*=146; a) and the PON-MAG dataset (*n*=142; b). The y-axis represents the partial effect (centered at zero) of each environmental variable on the expression index. Solid lines indicate fitted smoothing curves, dashed lines represent 95% confidence intervals, and open circles show partial residuals.

**Table 1.** Comparison of assembly statistics and BUSCO completeness between *de novo* transcriptomes and reference Parmales genomes.

Species and strain	Accession	Number of sequences	Busco Completeness (percentage)
NIES_2565	PRJDB4267	29,568	589/697 (84.5%)
CCMP1866	SAMN02740122	19,596	538/697 (77.2%)
RCC1657	SAMN02740258	21,571	464/697 (66.6%)
RCC208	SAMN02740123	23,225	473/697 (67.9%)
RCC2347	SAMN02740277	19,213	425/697 (61.0%)
*Triparma laevis* f. *inornata*	GCA_012489335.1	13,395	459/697 (65.9%)
*Tetraparma gracilis*	GCA_030267505.1	15,987	383/697 (54.9%)
*Triparma retinervis*	GCA_030267625.1	13,713	456/697 (65.4%)
*Triparma laevis* f. *longispina*	GCA_030267805.1	17,316	675/697 (96.8%)
*Triparma verrucosa*	GCA_030267905.1	14,735	680/697 (97.6%)
*Triparma strigata*	GCA_030267945.1	15,257	680/697 (97.6%)
*Triparma columacea*	GCA_030267965.1	14,430	667/697 (95.7%)
*Lepidoparma frigida*	GCA_030267985.1	12,273	662/697 (95.0%)

**Table 2.** Coefficients of determination (R^2^) between S-type and F-type marker abundance in metagenomic and metatranscriptomic datasets.

MAG	Dataset	Correlation (R^2^)
ARC-MAG	Metagenome	0.5875
ARC-MAG	Metatranscriptome	0.3721
PON-MAG	Metagenome	0.8143
PON-MAG	Metatranscriptome	0.5834
