# Supplementary material for: Metatranscriptome Data Support the Existence of Two Distinct Morphotypes in a Single Parmalean Species in Natural Environments
Source: Microbes Environ. 2026 Sep 18;41(3):ME26037. doi: 10.1264/jsme2.ME26037 (PMC13659343; doi:10.1264/jsme2.ME26037)
Supplement: Supplementary file 1 [file 41_26037_s1.pdf]

Supplementary figures

Metatranscriptome data support the existence of two distinct morphotypes in a single parmalean species in natural environments

Hiroto Sasaki[1], Hisashi Endo[1], Eric Pelletier[2,3], Shinya Yoshikawa[4], Akira Kuwata[5], Hiroyuki Ogata[1,*]

[1]Bioinformatics Center, Institute for Chemical Research, Kyoto University, Gokasho, Uji, Kyoto, 611-0011, Japan

[2]Génomique Métabolique, Genoscope, Institut François-Jacob, CEA, CNRS, Univ Evry, Université Paris-Saclay, F-91000 Evry, France

[3]Research Federation for the Study of Global Ocean Systems Ecology and Evolution, FRA2022/Tara GOSEE, F-75016 Paris, France

[4]Faculty of Marine Science and Technology, Fukui Prefectural University, Fukui, 917-0003, Japan

[5]Shiogama Field Station, Fisheries Resources Institute, Japan Fisheries Research and Education Agency, 3-27-5 Shinhama-cho, Shiogama, Miyagi, 985-0001, Japan

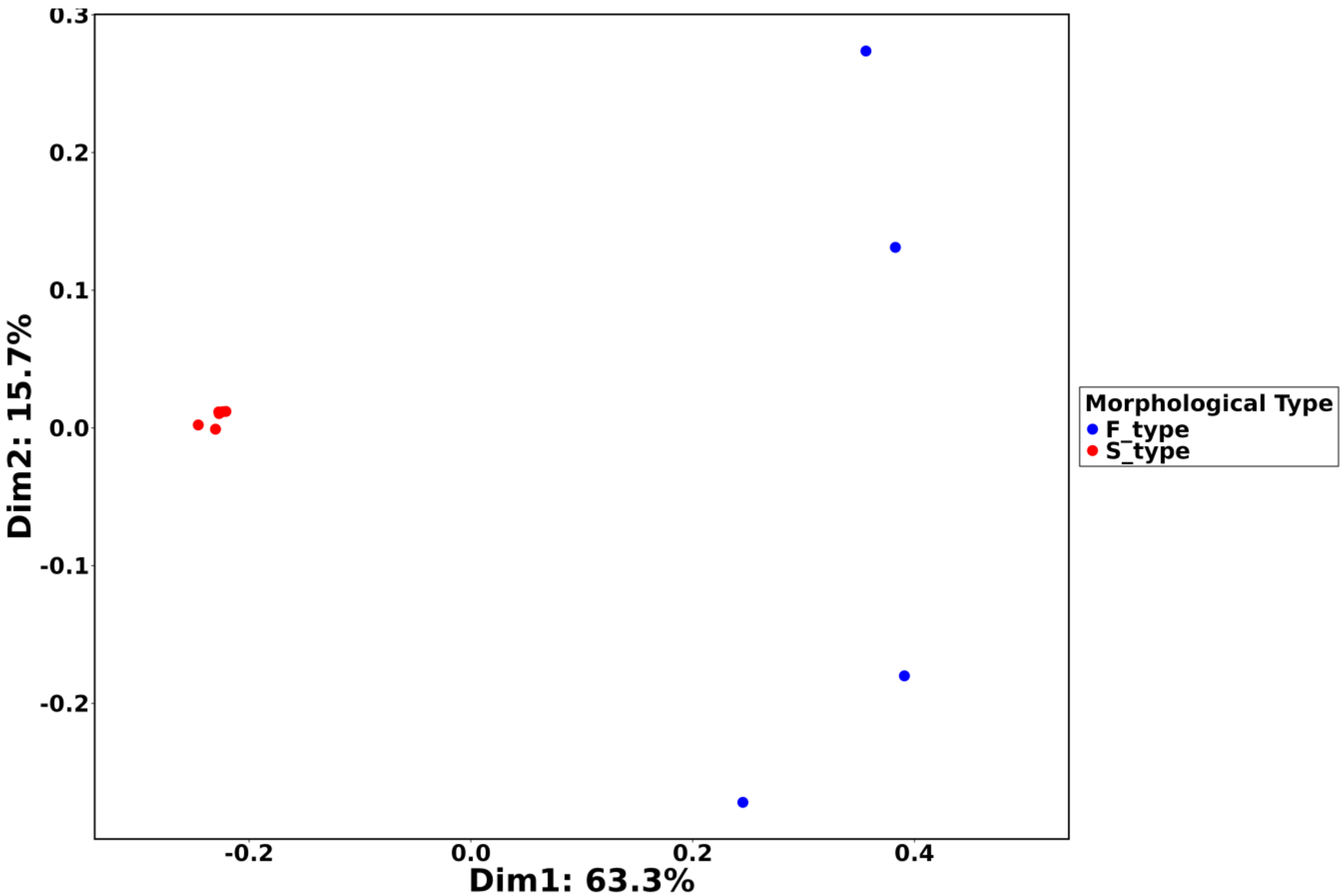

Figure S1. PCoA plot of gene expression profiles. The plot is based on Bray-Curtis dissimilarity calculated from read counts mapped to orthologous genes across 10 RNA-seq samples. Each point represents an individual RNA-seq sample. Blue points indicate the four F-type strains, while red points indicate the six S-type samples (including both silicate-rich and silicate-deficient conditions).

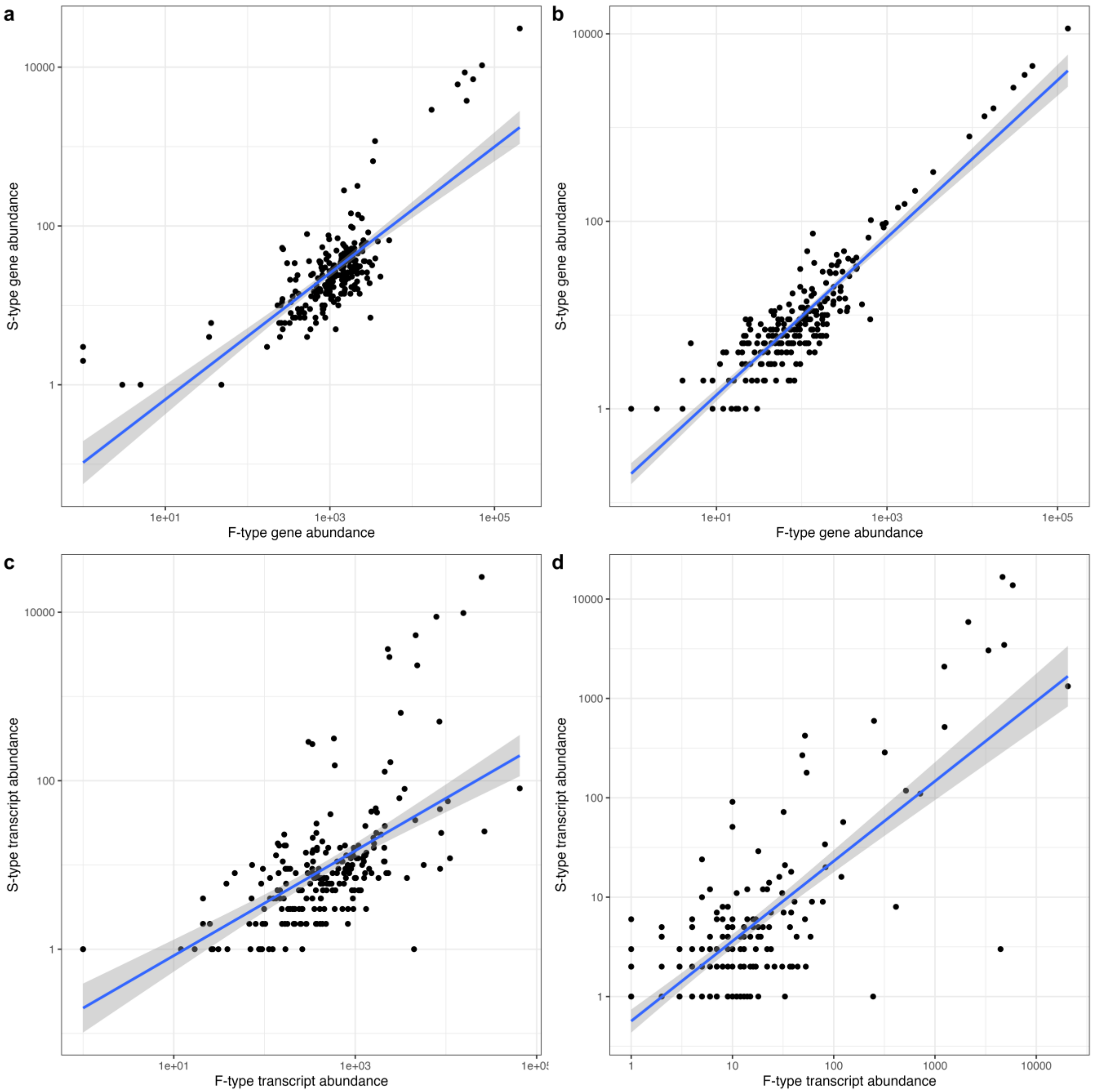

Figure S2. Comparison of genomic and transcriptomic abundance ratios between S- and F-type markers. Relationship between F-type and S-type marker abundances for ARC-MAG (a, c) and PON-MAG (b, d). (a, b) Metagenomic abundance showing consistent gene ratios across global sites. (c, d) Metatranscriptomic abundance showing variable transcript ratios, indicating differential expression. Each point represents a sampling site. Blue lines indicate linear regression fits with 95% confidence intervals (gray shading). Both axes are log-scaled.

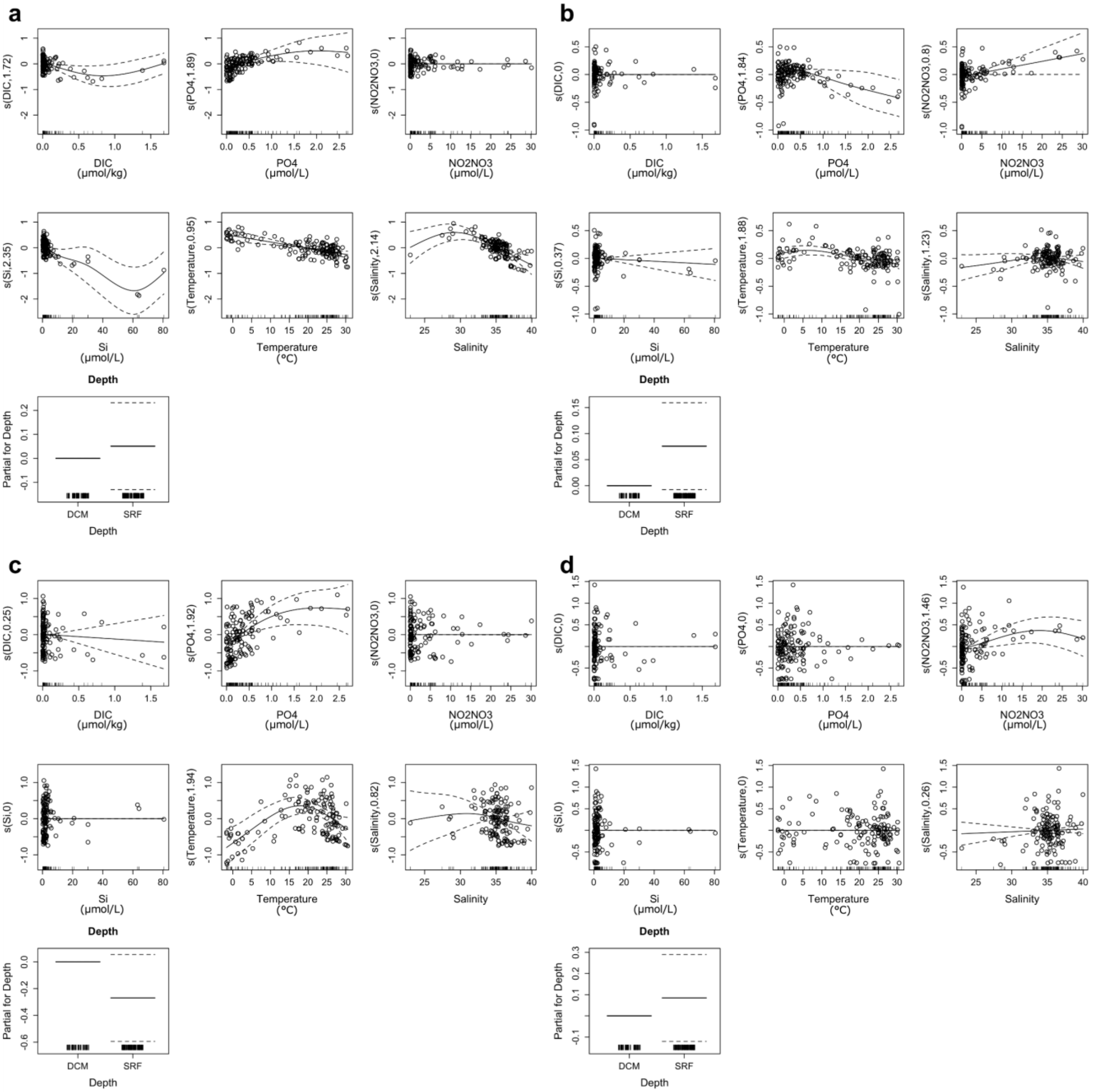

Figure S3. Generalized Additive Model (GAM) regression analysis of morphotype-specific expression variations based on environmental factors. Panels show partial effects of environmental variables on expression indices for the ARC-MAG dataset (n=146; a, b) and the PON-MAG dataset (n=142; c, d). Specifically, panels (a, c) correspond to S-type marker genes, while panels (b, d) correspond to F-type marker genes. The y-axis represents the partial effect (centered at zero) of each environmental variable on the expression index. Solid lines indicate the fitted smoothing curves, dashed lines represent 95% confidence intervals, and open circles show the partial residuals.
